# Visual Localization Domain for Accurate V-SLAM from Stereo Cameras

**DOI:** 10.3390/s25030739

**Published:** 2025-01-26

**Authors:** Eleonora Di Salvo, Sara Bellucci, Valeria Celidonio, Ilaria Rossini, Stefania Colonnese, Tiziana Cattai

**Affiliations:** Department of Information Engineering, Electronics and Telecommunications, Sapienza University of Rome, 00184 Rome, Italy; eleonora.disalvo@uniroma1.it (E.D.S.); tiziana.cattai@uniroma1.it (T.C.)

**Keywords:** visual localization, circular harmonic functions, visually relevant features, stereo camera

## Abstract

Trajectory estimation from stereo image sequences remains a fundamental challenge in Visual Simultaneous Localization and Mapping (V-SLAM). To address this, we propose a novel approach that focuses on the identification and matching of keypoints within a transformed domain that emphasizes visually significant features. Specifically, we propose to perform V-SLAM in a VIsual Localization Domain (VILD), i.e., a domain where visually relevant feature are suitably represented for analysis and tracking. This transformed domain adheres to information-theoretic principles, enabling a maximum likelihood estimation of rotation, translation, and scaling parameters by minimizing the distance between the coefficients of the observed image and those of a reference template. The transformed coefficients are obtained from the output of specialized Circular Harmonic Function (CHF) filters of varying orders. Leveraging this property, we employ a first-order approximation of the image-series representation, directly computing the first-order coefficients through the application of first-order CHF filters. The proposed VILD provides a theoretically grounded and visually relevant representation of the image. We utilize VILD for point matching and tracking across the stereo video sequence. The experimental results on real-world video datasets demonstrate that integrating visually-driven filtering significantly improves trajectory estimation accuracy compared to traditional tracking performed in the spatial domain.

## 1. Introduction

**Figure 1:**
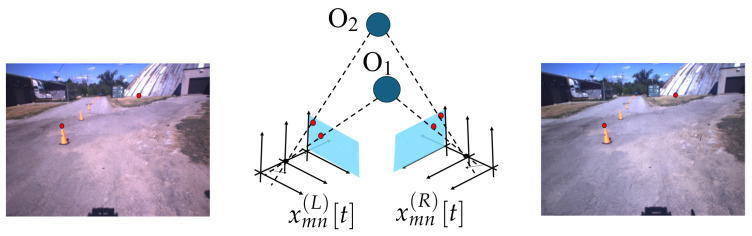
Acquisition geometry: Architecture and acquisition geometry, illustrating the capture of objects within the video scene. The diagram displays two frames, representing the left and right perspectives, highlighting the spatial relationship and overlapping fields of view between the frames. This setup is fundamental for analyzing depth, structure, and motion within the captured scene, showcasing the stereo alignment and relative orientation used during the recording process.

The ability to navigate and map unknown environments in real time is a crucial capability for autonomous systems [1,2]. Visual Simultaneous Localization and Mapping (VSLAM) enables devices such as robots, autonomous vehicles, and augmented reality (AR) platforms to achieve this by utilizing visual information from cameras to simultaneously construct a map of the environment while tracking their position within it [3,4]. As VSLAM technology has progressed, it has become increasingly important for dynamic, real-time applications, where systems must overcome challenges such as moving objects, fluctuating lighting conditions, and limited computational resources. For instance, in AR applications, SLAM enables the accurate overlay of virtual objects onto physical spaces, which requires precise localization and mapping under potentially difficult lighting or environmental conditions. Similarly, autonomous vehicles rely on SLAM to generate maps on-the-fly while adjusting to changes in the environment to ensure safe navigation [5]. SLAM methods have also advanced with improvements in sensor technology, processing power, and algorithmic techniques, which allow for higher accuracy and adaptability. However, real-world environments are rarely static and often demand sophisticated, adaptive SLAM solutions capable of handling dynamic conditions and external disturbances. These challenges underscore the need for innovative methods that can enhance SLAM’s robustness, accuracy, and efficiency in dynamic scenarios. In this work, we propose to perform simultaneous localization and mapping in a VIsual Localization Domain (VILD), i.e., a domain where visually relevant features are suitably represented for simultaneous localization and mapping (SLAM). To this aim, we consider a stereo camera acquisition system as illustrated in Figure 1, and we leverage the known properties of Fisher information to detect and recognize specific image patterns. Specifically, in [6], the authors demonstrate that transforming images into a domain defined by a basis of orthogonal Circular Harmonic Function (CHF) filters with specific radial profiles enables straightforward maximum likelihood localization of 2D patterns. In this domain, the maximum likelihood estimation of visual pattern translation and rotation is achieved using a quadratic loss function. Therefore, the output of Circular Harmonic Filters can be used as a meaningful domain for signal representation. The outputs from filters of different orders highlight visually relevant features. Furthermore, they appear directly in the maximum likelihood estimation of image transformation parameters, such as scale factors, rotation, or translation. Building on this, VILD-SLAM method adopts filtering based on two-dimensional CHF, leveraging both magnitude and phase information to refine feature localization and reduce key errors such as mean squared error and scale drift. The VILD-SLAM process consists of two primary stages:

- *Computation of VILD*: VILD highlights visually relevant regions. Specifically, after applying the CHF to detect high-intensity interest points corresponding to prominent structural edges in the environment, we compare the output magnitude against a threshold for feature localization. Then, we refine the output phase by selecting only the most relevant points, thus identifying the directions of visual structures.
- *VILD feature extraction and tracking*: This stage adopts VILD to identify abrupt changes of the local structure direction and use this information to extract keypoints to be used for tracking and localization.

This domain allows us to improve feature matching and tracking accuracy.

The incorporation of these filtering stages improves accuracy even with lower resolution images, such as those captured at larger distances. This has led to notable improvements in trajectory accuracy by aligning the estimated SLAM trajectory more closely with GPS data. The experimental results indicate that the proposed CHF-based method effectively reduces key errors, thereby providing a more accurate trajectory estimation and improved performance in dynamic environments.

## 2. Related Works

Traditional SLAM methods [7] generally rely on the assumption of static environments and utilize geometric techniques for localization and mapping, possibly exploiting application-specific constraints [8] or memory-efficient data representation [9]. While effective in controlled settings, these approaches often struggle with dynamic elements commonly encountered in real-world environments, such as moving objects or sudden lighting changes, as discussed in [10,11]. To overcome these limitations, recent research has focused on more adaptive SLAM methods that integrate artificial intelligence (AI), deep learning, and advanced hardware optimizations, enhancing SLAM’s robustness and accuracy in dynamic settings [12,13]. Systems like ORB-SLAM2 [14] and DFT-VSLAM [7] utilize advanced tracking techniques and dynamic feature extraction to improve performance in dynamic environments. Additionally, deep learning-based frameworks, such as AnyFeature-VSLAM [15], adaptively manage visual features across different scenarios, maintaining high accuracy and reliability (see [16] for a comprehensive survey).

VILD-SLAM advances the literature by leveraging Circular Harmonic Filters (CHFs) to improve feature detection and tracking, offering a robust alternative to existing methods. Unlike [17], which integrates points and lines in dynamic environments, VILD-SLAM approach focuses on CHF coefficients for noise-robust edge and orientation detection, optimizing trajectory accuracy. Similarly, while [18] introduces planar constraints for road-based SLAM, VILD-SLAM excels in extracting image transformations under varying conditions, enhancing adaptability. By refining stereo feature alignment compared to standard methods, VILD-SLAM complements and extends insights from [19] and trajectory evaluations in [20].

## 3. System Model

**Figure 2:**
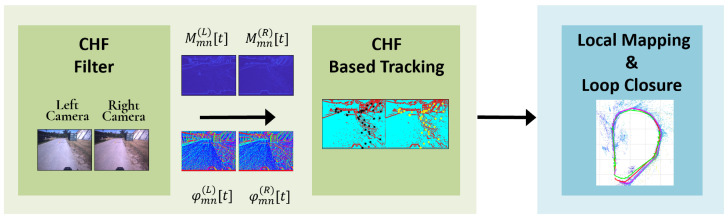
The figure illustrates the pipeline system, from acquiring the stereo video sequence pair to the final output: the estimated trajectory, possibly combined with a 3D representation of the ambient. Three main steps can be identified: visual feature extraction using the CHF filter, and CHF-based tracking, followed by SLAM procedures for data mapping and possible loop closure in case the acquisition returns to previously observed areas.

Herein, we consider the system outlined in Figure 2. The acquisition and tracking step reflect the architecture in [14]. The acquisition is a stereo-type setup that relies on two sensors: a left camera and a right camera. The input video sequences are denoted as

(1) $$I_{mn}^{\left(L\right)}\left[t\right],\;I_{mn}^{\left(R\right)}\left[t\right],m\;=\;0,\cdots M\;-\;1,n\;=\;0,\cdots N\;-\;1,t\;=\;0,\cdots$$

where M×N is the sequence spatial resolution and *t* represents the *t*-th temporal index of the video.

The input video sequences are then represented into a transformed domain, by CHF filtering, as detailed in Section 3, using the notation summarized in Table 1. At each frame, two primary steps are performed. The first is visual feature extraction, where the proposed CHF filtering extracts structural information from the image using a complex representation that includes magnitude and phase components. This enhances the identification and tracking of keypoints, improving accuracy, as demonstrated in experimental results. The second step is localization and mapping, where a 3D point map is initialized from a stereo image disparity map. Features are then tracked and updated, potentially recalculating disparity until revisiting a previously explored position in the environment.

**Table 1:** Main notation.

Notation	Description
$I_{mn}^{\left(L\right)}\left[t\right],I_{mn}^{\left(R\right)}\left[t\right]$	Original Left and right images at time t,t=0,⋯
$h_{mn}^{\left(l\right)}$	Impulse response of the visual feature extraction filter
${\hat{I}}_{mn}^{\left(L\right)}\left[t\right],{\hat{I}}_{mn}^{\left(R\right)}\left[t\right]$	Complex images at the output of the filter $h_{mn}^{\left(l\right)}{|}_{l\;=\;1}$
$M_{mn}^{\left(L\right)}\left[t\right],M_{mn}^{\left(R\right)}\left[t\right]$	Module of ${\hat{I}}_{mn}^{\left(L\right)}\left[t\right],{\hat{I}}_{mn}^{\left(R\right)}\left[t\right]$
$\varphi_{mn}^{\left(L\right)}\left[t\right],\varphi_{mn}^{\left(R\right)}\left[t\right]$	Phase of ${\hat{I}}_{mn}^{\left(L\right)}\left[t\right],{\hat{I}}_{mn}^{\left(R\right)}\left[t\right]$
$\psi_{mn}^{\left(L\right)}\left[t\right]\psi_{mn}^{\left(R\right)}\left[t\right]$	Phase threshold based on the magnitude components

## 4. Visual Features in the CHF Domain: A Review

The literature has shown that the output of Circular Harmonic Filters can be used as a meaningful domain for signal representation. The outputs from filters of different orders highlight visually relevant features. Furthermore, they appear directly in the maximum likelihood estimation of image transformation parameters, such as scale factors, rotation, or translation. In this section, we review the theory, while in the next, we explain how to apply it to the matching and tracking of points between the right and left sequences of a stereo video sequence.

The extraction of visual features has been widely applied in image processing, because it can detect representative image features, such as edges, lines, and intersections.

This procedure provides valuable information about the structures of the output image; it highlights edges while simultaneously measuring their intensity (magnitude) and direction (orientation). Among others, Circular Harmonic Filters (CHFs), formerly introduced in a previous study, exhibit theoretical properties related to how they characterize the information associated with the visually relevant structure.

The mathematical formulation of CHFs, and their impact for visual feature extraction and tracking is presented below.

Let us recall the definition of the CHF in a 2D continuous domain. In polar coordinates (*r*, θ), representing the distance from the origin and the angle concerning the *x*-axis, respectively, the CHF of order *k* is the complex filter defined by the Formula (2):

(2) $$h^{\left(k\right)}\left(r,\theta \right)=L^{\left(k\right)}\left(r\right)e^{jk\theta }$$

The functions $L^{\left(k\right)}\left(r\right)$ in Equation (2) are isotropic Gaussian-weighted kernels, known as Laguerre–Gauss functions:

$$L^{\left(k\right)}\left(r\right)=r^{k}e^{-\frac{r^{2}}{a_{k}}}$$

which satisfy isomorphism with the frequency space. The variable *k* defines the angular structure of the model: for k=0, the CHF output is a low-pass version of the input image. As the order *k* increases, CHFs highlight increasingly complex directional structures in the visual data, such as edges (for k=1), lines (for k=2), bifurcations (for k=3), and intersections (for k=4). In the followings, we refer to first-order CHF (l=1), which has a band-pass behaviour. In the frequency domain, the following formula, Formula (3) stands:

(3) $$|H^{\left(k\right)}\left(\omega_{1},\omega_{2}\right)|={\left(\sqrt{\omega_{1}^{2}+\omega_{2}^{2}}\right)}^{k}\cdot \frac{a_{k}}{N}\cdot e^{\frac{1}{2}{\left(\frac{a_{k}}{N}\right)}^{2}\left(\omega_{1}^{2}+\omega_{2}^{2}\right)},\;$$

with $\omega_{1},\omega_{2}\;=\;0,2\pi /N,\cdots 2\pi \left(N-1\right)/N$. The module $|H{\left(\omega_{1},\omega_{2}\right)}^{\left(k\right)}|$ results from the product of two factors: a radial factor, corresponding to a derivative action, and a Gaussian low-pass factor. The phase is written as follows:

(4) $$∠H\left(\omega_{1},\omega_{2}\right)=k\cdot \arctan_{2}\left(\omega_{1},\omega_{2}\right)$$

**Figure 3:**
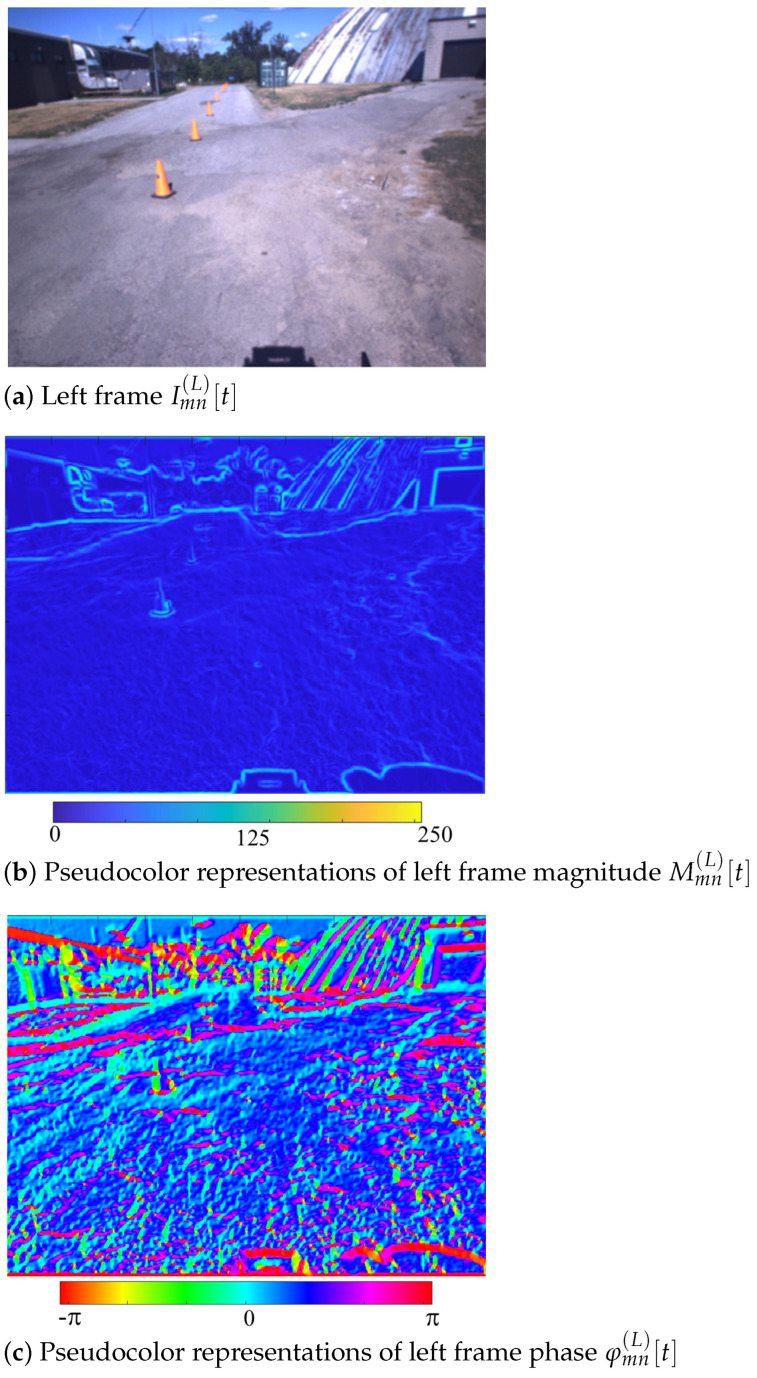
Original frame (**a**), magnitude $M_{mn}^{\left(L\right)}\left[t\right]$ (**b**) and phase $\varphi_{mn}^{\left(L\right)}\left[t\right]$ (**c**) of the CHF output at t=0.

The CHF filter output is meaningful in revealing the visually relevant structure in an image. A visual example using the first-order CHF (k=1) on a real image $I_{mn}^{\left(L\right)}\left[t\right]$ at k=0 is shown. Figure 3 (top) represents the original frame before the application of the CHF filter, while in Figure 3 (bottom) we report the magnitude $M_{mn}^{\left(L\right)}\left[t\right]$ and the phase $\varphi_{mn}^{\left(L\right)}\left[t\right]$ of the CHF output. Notably, the magnitude highlights the strength of the edge and the phase identifies its orientation. Recently, the CHF filter has been extended to the non-Euclidean domain, over manifolds such as those underlying point-cloud data [21].

The CHF filters play also a relevant role in maximum likelihood (ML) estimation of translation, scaling, and rotation parameters for natural images, as demonstrated in [6]. The rational behind this is as follows. Let $g_{mn}$ denote a reference image, and let $g_{mn}$ be observed in presence of a scaling by a factor α, a rotation by an angle β, and a translation by a displacement Δ. Let us denote by

$$f_{mn}=T\left\{g_{mn};\alpha ,\beta ,\Delta \right\}+w_{mn}$$

the observation of the transformed version of $g_{mn}$ in presence of an additive white Gaussian noise independent on $g_{mn}$. The ML estimate of the parameters α,β,Δ is obtained by maximizing the log-likelihood function $\Lambda (\alpha ,\beta ,\Delta |f_{mn})$ of the observed image $f_{mn}$ with respect to the unknown parameters, i.e.,:

$$\left({\hat{\alpha }}_{ML},{\hat{\beta }}_{ML},{\hat{\Delta }}_{ML}\right)=\arg\max_{\alpha ,\beta ,\Delta }\Lambda \left(\alpha ,\beta ,\Delta |f_{mn}\right)$$

In white Gaussian noise, the ML estimate are directly obtained by minimizing Euclidean distance

$$\left({\hat{\alpha }}_{ML},{\hat{\beta }}_{ML},{\hat{\Delta }}_{ML}\right)=\arg\min_{\alpha ,\beta ,\Delta }\left|\right|f_{mn}-T\left\{g_{mn};\alpha ,\beta ,\Delta \right\}{\left|\right|}^{2}$$

The minimization of the Euclidean distance can also be obtained in a transformed orthonormal space. Let us recall the following mathematical results:

(i) The development of a generic function u(r,θ) in a series of orthonormal polar basis functions [6,22] as:
  $$u\left(r,\theta \right)=\sum_{n}u_{n}\left(r\right)e^{jn\theta },$$
  being $u_{n}\left(r\right)$ the Fourier transform $u_{n}\left(r\right)=F_{\theta }\left\{u\left(r,\theta \right)\right\}$ and
(ii) The development of $u_{n}\left(r\right)$ in a series of Laguerre–Gauss functions [6,23]
  $$u_{n}\left(r\right)=\sum_{k}U_{k}^{\left(n\right)}L^{\left(k\right)}\left(r\right).$$

The application of these results in the discrete domain allows us to compute the ML cost function in a transformed space. As shown in [6], the ML estimate of the parameters α,β,Δ is found by minimizing the distance of the transformed coefficients of the observed image $f_{mn}$ and the transformed version of the template $T\{g_{mn};\alpha ,\beta ,\Delta \}$. These coefficients can in turn be obtained at the output of CHF filters of different orders.

## 5. Visual Localization Domain

Expanding on the above-described properties, to build the Visual Localization Domain, we resort to a first-order approximation of the image-series representation, computing the first-order coefficients directly through the application of the first-order CHF. This corresponds to representing the image in a theoretically grounded, visually relevant domain. We leverage this domain for point matching and tracking over the stereo video sequence. We have seen that Circular Harmonic Filters (CHFs) enable a visually relevant representation of signals while simultaneously providing a domain where the maximum likelihood estimation of signal parameters can be directly achieved by minimizing a quadratic distance in the coefficient domain. Here, we focus solely on the coefficients’ output by the first-order filter. For the point matching and tracking problem under consideration, we can assume that one of the stereo views serves as the reference for matching, while the goal is to identify the most similar version in the other view. This search is conducted not in the original domain but in the coefficient domain of the filter output. These coefficients allow for the identification of parameters such as rotation, as well as translation and scale, in terms of minimal mean squared error. As a result, they facilitate the search for similarities under such local image transformations. Furthermore, they are inherently robust to noise due to the low-pass effect typical of the Gaussian profile at high frequencies. The procedure resulting from these considerations is described below.

Applying the CHF filtering to the input sequences generates two complex sequences

$$\left\{\begin{array}{c} {\hat{I}}_{mn}^{\left(L\right)}\left[t\right]=I_{mn}^{\left(L\right)}\left[t\right]\;*\;h_{mn}^{\left(k\right)}\\ {\hat{I}}_{mn}^{\left(R\right)}\left[t\right]=I_{mn}^{\left(R\right)}\left[t\right]*h_{mn}^{\left(k\right)} \end{array}\right.\;$$

obtained by convolving the luminance of the left and right original images with the impulse response $h^{\left(k\right)}\left(r,\theta \right)$. Therefore, the filtered images are characterized in terms of the modules and phase $M_{mn}^{\left(L\right)}\left[t\right],\;M_{mn}^{\left(R\right)}\left[t\right]$, $\varphi_{mn}^{\left(L\right)}\left[t\right],\;\varphi_{mn}^{\left(R\right)}\left[t\right]$. The function [6,24] returns the magnitude and phase of the filtered image, which are useful for extracting the edges of objects present in the reference scene.

The outcome is a complex image in which each edge is associated with a high amplitude value of the magnitude, while the phase provides useful information on the spatial directions of the visually relevant image components. In contrast, uniform areas correspond to low-intensity and pseudo-random phase values [6,24]. Therefore, it can be stated that the CHF filter emphasises the presence of edges and measures their strength and orientation.

The next phase involves applying the following procedure, developed as follows. Firstly, the histogram $\hat{p}\left(\mu \right)$ of the normalised magnitude

$$\mu_{mn}^{\left(L\right)}\left[t\right]=\frac{M_{mn}^{\left(L\right)}\left[t\right]}{\max_{m,n}M_{mn}^{\left(L\right)}\left[t\right]}\;$$

at t=0 is computed. Since the magnitude highlights selected regions (edges), the histogram is typically multimodal, with one peak representing real edges and a second peak, near zero, representing high-frequency noise components. Hence, the areas relevant for point extraction and tracking can be highlighted by suitably selecting a threshold value $M_{0}$ on the magnitude $M(\omega_{1},\omega_{2})$. Specifically, the relevant areas are obtained as the set of points such that

$$\;\mu_{mn}^{\left(L\right)}\left[t\right]>M_{0}\;$$

which means that it is nonzero only at frequencies where the normalized magnitude values are above the threshold.

We improve the estimate of the orientation information by updating the phase based on the magnitude, and in particular computing the stereo visual phase sequences $\psi_{mn}^{\left(L\right)}\left[t\right],\psi_{mn}^{\left(R\right)}\left[t\right]$:

$$\left\{\begin{array}{c} \psi_{mn}^{\left(L\right)}\left[t\right]=\varphi_{mn}^{\left(L\right)}\left[t\right],M_{mn}^{\left(L\right)}\left[t\right]\;>\;M_{0};\;0\;\mathrm{otherwise}\\ \psi_{mn}^{\left(R\right)}\left[t\right]=\varphi_{mn}^{\left(R\right)}\left[t\right],M_{mn}^{\left(R\right)}\left[t\right]\;>\;M_{0};\;0\;\mathrm{otherwise} \end{array}\right.$$

**Figure 4:**
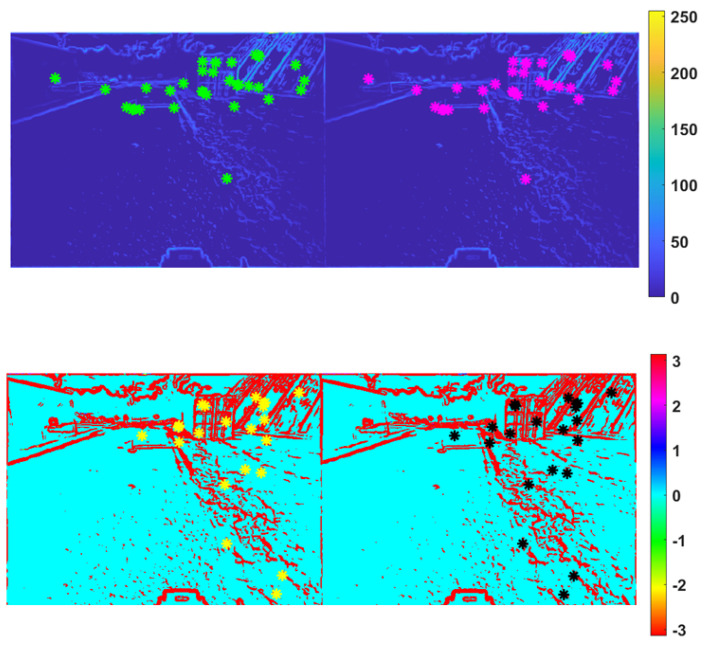
Results of threshold application (θ=0.1) on frame 100 after CHF: matched features of magnitude (**top**), and matched features of phase (**bottom**).

An example of feature extracted at the output of the CHF filter appears in Figure 4, showing the matched features on the magnitude maps $M_{mn}^{\left(L\right)}\left[t\right],M_{mn}^{\left(R\right)}\left[t\right]$ and the phase maps $\varphi_{mn}^{\left(L\right)}\left[t\right],\varphi_{mn}^{\left(R\right)}\left[t\right]$ (bottom) for frame 100 (θ=0.1). Although meaningful, the magnitude map reports just an edge intensity information, while the phase map is rather noise. These limitations are overcome by the VILD maps $\psi_{mn}^{\left(L\right)}\left[t\right],\psi_{mn}^{\left(R\right)}\left[t\right]$.

**Figure 5:**
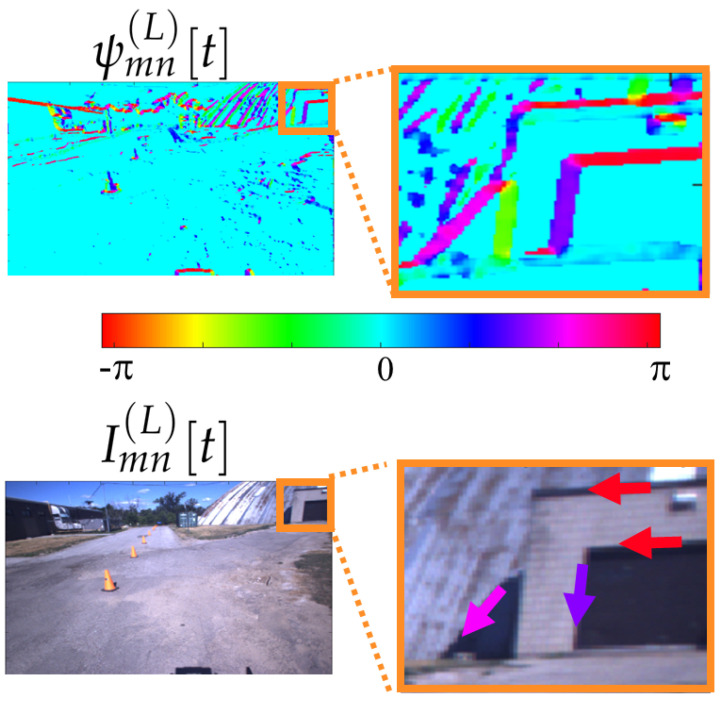
A visual representation of the meaning associated to $\psi_{mn}^{\left(L\right)}\left[t\right]$: the map is different from zero only in correspondence of structured areas, and the value at each (m,n) pair represents the direction of the edge at the corresponding pixel in $I_{mn}^{\left(L\right)}\left[t\right]$.

An interpretation of the role of $\psi_{mn}^{\left(L\right)}\left[t\right],\psi_{mn}^{\left(R\right)}\left[t\right]$ is provided in Figure 5, where we recognize that $\psi_{mn}^{\left(L\right)}\left[t\right]$ is different from zero only in the correspondence of structured areas, and the value at each (m,n) pair represents the direction of the edge at the corresponding pixel in $I_{mn}^{\left(L\right)}\left[t\right]$.

### Remarks

A few remarks are in order. Firstly, VILD computational complexity is very low, since it reduces to filtering and thresholding techniques that can be realized using hardware acceleration or parallel multi-core processing on different image regions. Specifically, VILD requires a filtering operation, and the calculation of VILD introduced an additional complexity of

$$\Delta C\approx \underset{FFT/IFFT}{\underset{︸}{2N^{2}log\left(N\right)}}+\underset{Filter}{\underset{︸}{\rho \left(a_{1}\right)N^{2}}}$$

to realize the filtering of an N×N image in the Fourier domain, where, due to the band-pass nature of CHF filtering, only a fraction $\rho (a_{1})<1$ of coefficients have to be calculated; real-time solutions for this kind of computation are available in the literature [25].

In addition, VILD is determined based on two parameters, namely the first order CHF filter parameter $a_{1}$ and the threshold θ. The first can be *a priori*, assigned by selecting its value relative to the resolution, while the threshold can be selected by histogram analysis of the CHF output module.

Finally, herein we have adopted VILD within a recursive algorithm. Still, the approach behind VILD is very general, and can be generalized also to deep learning approaches for VSLAM purposes, such as [26,27]. In particular, the deep learning algorithm can be applied directly in the VILD domain, provided careful training and hyperparameter optimization is provided. Notice that, in principle, VILD is not sufficient for the full reconstruction of images in the original domain, since at least a low-pass representation as that provided by Parameter’s estimation 0-th order CHF filtering (Gaussian filtering) is needed. Then, VILD representation can be provided to the deep neural network in parallel with images represented in the spatial domain. This relevant development is left for future studies.

## 6. Experimental Results

The effectiveness of the VILD-SLAM algorithm was evaluated using real-world stereo camera datasets. We present results of VSLAM in the VILD domain, based on the sequences $\psi_{mn}^{\left(L\right)}\left[t\right]$ and $\psi_{mn}^{\left(R\right)}\left[t\right]$. For comparison, we report also results of the state-of-the-art method in [14], in the implementation available at [28], and the results obtained when performing tracking on $M_{mn}^{\left(L\right)}\left[t\right],M_{mn}^{\left(R\right)}\left[t\right]$.

**Figure 6:**
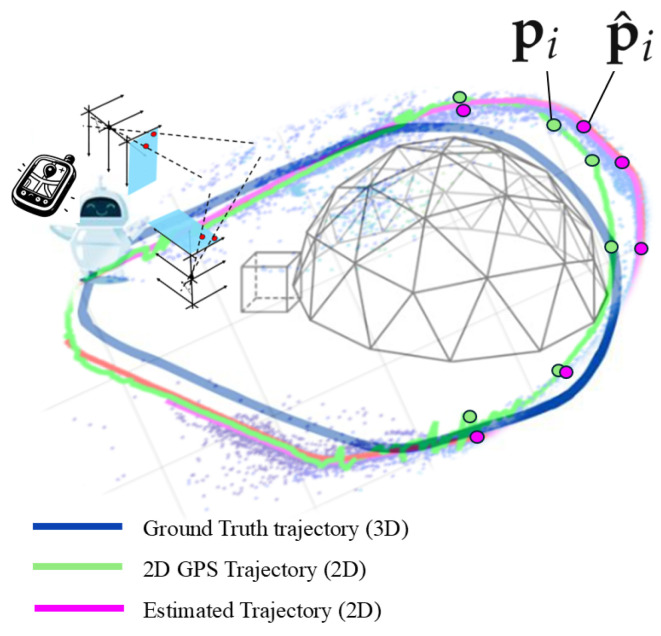
The selected stereo video sequences belong to the dataset [29], and were captured by a robot equipped with stereo cameras and with a GPS device. The robot follows a closed loop. The true trajectory follows a 3D path; the 2D GPS trajectory, disregarding changes in altitude, is assumed as a ground truth for VSLAM algorithm validation by comparison of the GPS location and the one estimated by V-SLAM over a set of $N_{p}$ reference points.

The experiments use a stereo video sequence from the dataset in [29]. These datasets consist of 1073 stereo image pairs, captured in July under sunny conditions. The selected frames, relative to the 5-th run, are used to construct and compare trajectories. Following a training phase, a robot equipped with a stereo camera autonomously traversed a 160-m route, in a natural landscape with occasional artificial structures, over repeated runs (see Figure 6). Stereo keyframes, defined as the frames containing a sufficient number of valid keypoints for mapping purposes, were recorded approximately every 0.2 m. The true trajectory follows a 3D path, while the GPS trajectory and the estimated trajectory refer to a 2D projection. It is important to note that the GPS trajectory is known to be affected by estimation errors, resulting in random fluctuations. However, since these errors are typically smaller than those affecting the V-SLAM algorithm, we will consider the 2D GPS trajectory—disregarding changes in altitude—as the ground truth for validating the V-SLAM algorithm. Validation is performed by comparing the GPS locations with the locations estimated by V-SLAM across a set of $N_{p}$ keypoints, which are assumed to be reference points.

The stereo camera configuration features a 0.24 m baseline and a resolution of 512×384. Images are captured at 16 Hz, with the full stereo stream subsequently downsampled to extract stereo keyframes at intervals of approximately 0.2 m traveled [29]. The simulation parameters are as follows: (i) the maximum horizontal displacement between corresponding keypoints was limited to 48 pixels, equating to 9.38% of the image width; (ii) the image pyramid employed a scale factor of 1.1 for size reduction; (iii) the pyramid included 10 levels.

**Figure 7:**
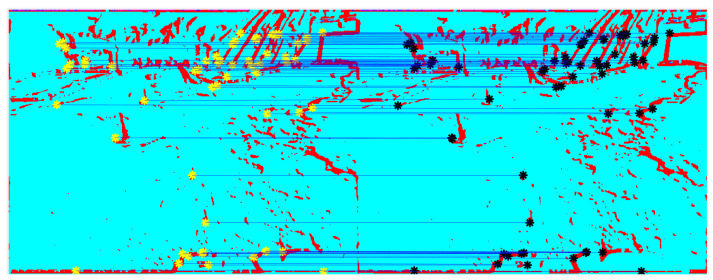
Juxtaposed VILD maps $\psi_{mn}^{\left(L\right)}\left[t\right]$, $\psi_{mn}^{\left(R\right)}\left[t\right]$ observed at the output of the CHF application, and keypoints matches at frame t=0.

Building on the framework outlined above, we assess the accuracy of the trajectory estimation compared to GPS benchmarks using VILD-based VSLAM. Applying the CHF filter to the luminance channel of the original images leads to the magnitude and phase components $M_{mn}^{\left(L\right)}\left[t\right],M_{mn}^{\left(R\right)}\left[t\right]$, and $\varphi_{mn}^{\left(L\right)}\left[t\right],\varphi_{mn}^{\left(R\right)}\left[t\right]$ enhancing edges and their direction, from which we compute the VILD maps $\psi_{mn}^{\left(L\right)}\left[t\right],\psi_{mn}^{\left(R\right)}\left[t\right]$, where keypoints are well concentrated in visually relevant regions. In Figure 7 we show the VILD maps $\psi_{mn}^{\left(L\right)}\left[t\right]$, $\psi_{mn}^{\left(R\right)}\left[t\right]$, and keypoints matches at frame t=0.

Stemming from these calculations, the tracking is then performed in the VILD domain. To evaluate the error between the estimated and GPS trajectories, two performance metrics are used: mean square error (MSE), scale drift (SD).

The MSE quantifies the average Euclidean distance, on the (x,y) plane, between the $N_{p}\times 2$ matrix P, collecting the $N_{p}$ reference key data points $p_{i}$ obtained by the GPS trajectories and the $N_{p}\times 2$ matrix $\hat{P}$ of their estimated counterparts ${\hat{p}}_{i}$. The MSE is computed as: $\mathrm{MSE}=\frac{1}{N_{p}}||P-\hat{P}{\left|\right|}_{F}^{2}=\frac{1}{N_{p}}\sum_{i=1}^{N_{p}}\left|\right|p_{i}-{\hat{p}}_{i}{\left|\right|}^{2}$. The scale drift SD, a secondary metric, measures systematic scale deviations between estimated and GPS trajectories. It is defined as a function of the scale factor $S_{HT}$ estimated through Helmert transformation, i.e., the scale factor computed as:

$$\mathrm{SD}=S_{HT}-1,\;S_{HT}=\arg\min_{s}||P-sR\hat{P}+T{\left|\right|}^{2}$$

quantifies the systematic deviation of the estimated trajectory from the true GPS trajectory: negative ($S_{HT}<1$) or positive SD values ($S_{HT}>1$) indicate the need for compression or expansion to align the estimated trajectory with the GPS path.

Table 2 reports the MSE of the VILD-SLAM based on the maps $\psi_{mn}^{\left(L\right)}\left[t\right],\psi_{mn}^{\left(R\right)}\left[t\right]$. The metrics shown are the mean square error (MSE [m^2^]), root mean square error (RMSE [m]) and scale drift (SD[]). In addition to the results for the complete path, metrics are provided for the first segment (from the beginning of the path to the end of the curve) and the second segment (from the end of the curve to the closure of the path). This segmentation enables a more precise assessment of VILD-SLAM performance. Transitory keypoints, i.e., points for which a match is found on less then $N_{M}$ consecutive frames, are discarded [14]. We report results for two different values of the threshold θ, namely $\theta \;=\;0.1,N_{M}\;=\;20$ and $\theta \;=\;0.08,N_{M}\;=\;5$. For the sake of comparison, in Table 3, we report the same metrics for the estimates obtained by the stereo ORB2-VSLAM algorithm in [14]. The VILD-SLAM shows improved performances with respect to the literature in both conditions.

**Figure 8:**
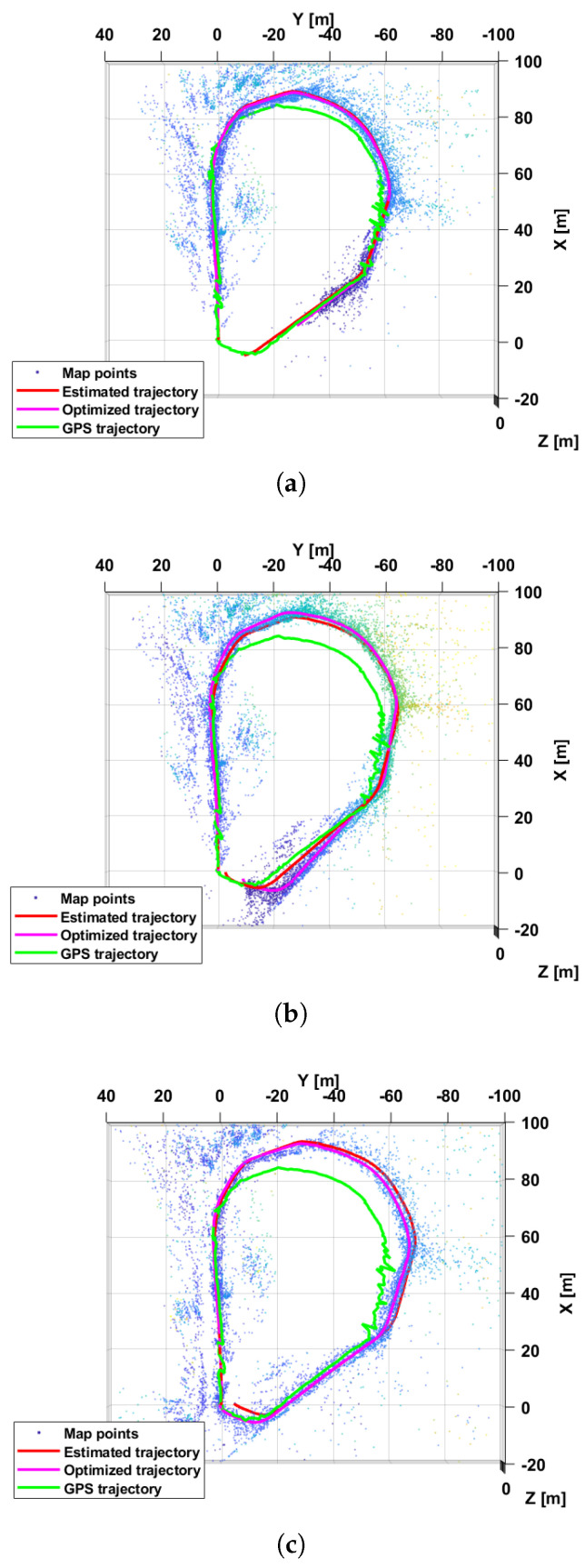
(**a**) Estimated and optimized trajectories (red, pink, respectively) obtained by VILD-SLAM operating on $\psi_{mn}^{\left(L\right)}\left[t\right],\psi_{mn}^{\left(R\right)}\left[t\right]$ with $\theta \;=\;0.1,N_{M}\;=\;20$ and (**b**) $\theta \;=\;0.08,N_{M}\;=\;5$, and ground-truth GPS trajectory (green). For the sake of comparison the (**c**) estimated and optimized trajectories (red, pink) obtained by the state-of-the-art algorithm in [14] are also reported.

Figure 8 illustrates the performance achieved by VILD-SLAM operating on $\psi_{mn}^{\left(L\right)}\left[t\right],\psi_{mn}^{\left(R\right)}\left[t\right]$ showing the ground-truth GPS (green), estimated and optimized trajectories (red, pink, respectively). The estimated 3D key points locations are also represented by colored dots [30,31]. The figure refers to the case of $\theta \;=\;0.1,N_{M}\;=\;20$ and $\theta \;=\;0.08,N_{M}\;=\;5$, respectively. For the sake of comparison, the trajectories obtained by the state-of-the-art algorithm in [14] are also reported.

**Figure 9:**
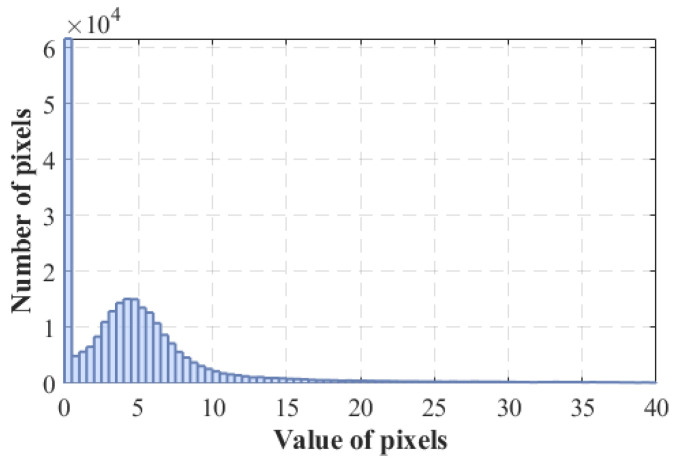
Histogram for threshold value evaluation: the abscissa reports the values assumed by $M_{mn}^{\left(L\right)}\left[t\right]$ at t=0, while the ordinate represents the value occurrences. We recognize the typical bimodal structure, with small values corresponding to noisy components in flat image areas while large values correspond to sparse image structures.

Implementing the CHF filter yielded both the magnitude and phase of the filtered image, followed by the magnitude thresholding and computation of the VILD maps $\psi_{mn}^{\left(L\right)}\left[t\right],\psi_{mn}^{\left(R\right)}\left[t\right]$. The threshold can be selected by analysis of the magnitude histogram, illustrated in Figure 9. We recognize the typical bimodal structure, with small values corresponding to noisy components in flat image areas, while large values correspond to sparse image structures.

**Figure 10:**
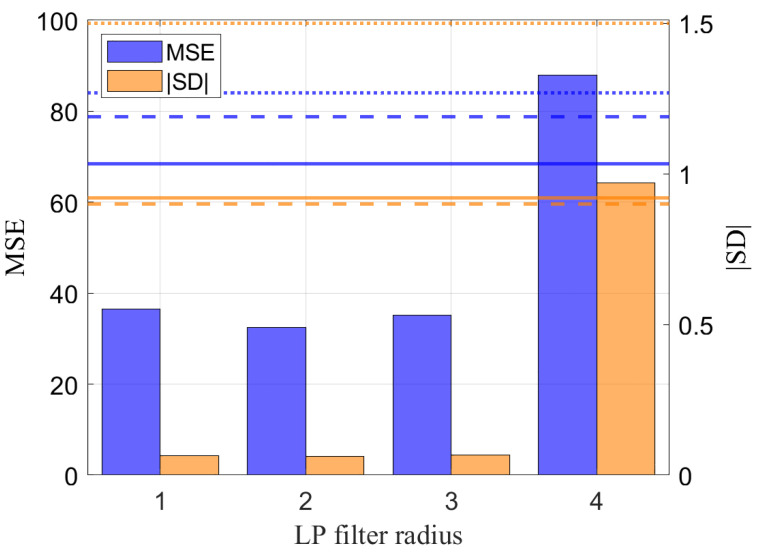
Mean squared error (MSE, left axis) and scale drift (SD, right axis) as a function of the low-pass (LP) filter radius. The blue bars represent the MSE, while the orange bars correspond to the absolute value of SD (|SD|). The horizontal lines indicate the reference MSE and |SD| values for the baseline ORB2-VSLAM method without any degradation; specifically, the solid, dashed, and dotted lines refer to the performance achieved using ORB, BRISK and SIFT features. This figure demonstrates the relationship between filter radius and trajectory performance, highlighting the impact of the image spatial bandwidth on the method accuracy.

A further analysis is conducted by degrading the stereo video sequences using a moving average filter defined over a circular support of radius R=1,2,3,4. This condition is taken as a proxy of a reduced spatial resolution condition, such as that encountered when the video sequence are acquired at a larger distance from the scene, or in harsh acquisition conditions, e.g., rain. The accuracy of VILD-SLAM using $\psi_{mn}^{\left(L\right)}\left[t\right],\psi_{mn}^{\left(R\right)}\left[t\right]$ is evaluated terms of the MSE and SD performance metrics. Figure 10 presents the MSE and SD as a function of the low-pass filter radius. The blue bars represent the MSE, while the orange bars correspond to the absolute value of SD (|SD|). The algorithm in [14] could not complete the analysis, even for different parameter settings, due to the increased difficulty in valid keypoint identification in the presence of image blur. For the sake of comparison, we report the MSE and |SD| values for the algorithm operating on the original, unblurred sequence, indicated by the horizontal lines. Specifically, we reported the results obtained by using the ORB-VSLAM2 method with BRISK features and SIFT features [32,33]. This figure demonstrates that the adoption of VILD for tracking also enables VSLAM on blurred images, maintaining meaningful trajectory estimations at different radii.

**Figure 11:**
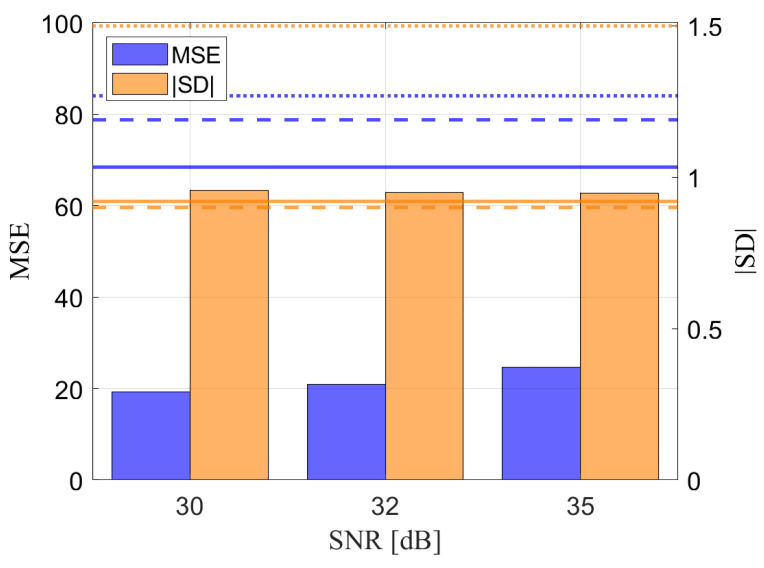
Mean squared error (MSE, left axis) and scale drift (SD, right axis) as a function of the SNR(dB). The blue bars represent the MSE, while the orange bars correspond to the absolute value of SD (|SD|). The horizontal lines indicate the reference MSE and |SD| values for the baseline ORB2-VSLAM method without any degradation; specifically, the solid, dashed, and dotted lines refer to the performance achieved using ORB, BRISK, and SIFT features, respectively. This figure demonstrates the relationship between SNR and trajectory estimation performance.

We now assess the performance of VILD-SLAM in noisy conditions, specifically when the images are acquired under an additive white Gaussian noise. Figure 11 shows the mean squared error (MSE, left axis) and scale drift (SD, right axis) as a function of the SNR(dB). To better frame the performances of VILD-SLAM, we also reported the results obtained by using the ORB-VSLAM2 method with BRISK features and SIFT features [32,33]. We recognize that it outperforms the state-of-the-art competitors. All together, the results show the improvement of VILD in terms of accuracy and resilience.

**Figure 12:**
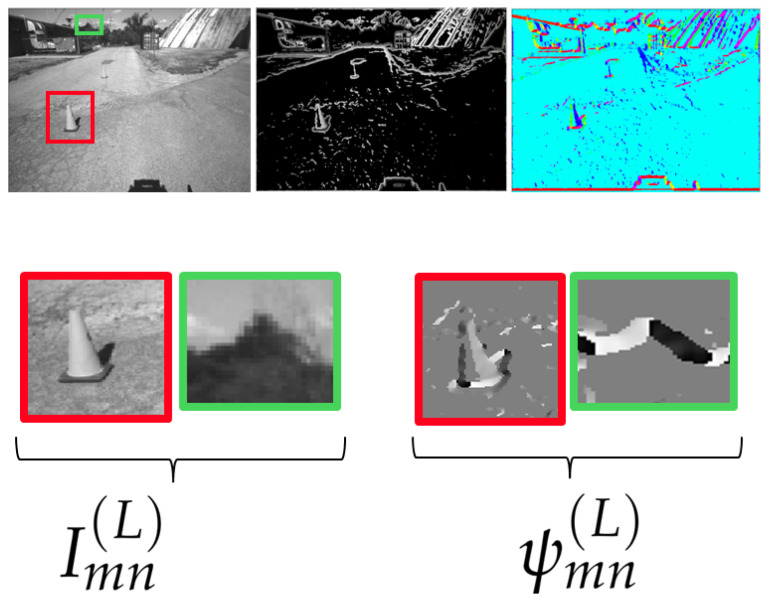
The figure illustrates: (first row) the original image $I_{mn}^{\left(L\right)}\left[t\right]$, the CHF output module $M_{mn}^{\left(L\right)}\left[t\right]$ and the thresholded phase $\psi_{mn}^{\left(L\right)}\left[t\right]$ (t=30), and (second row) the details of the captured image in the red and green boxes as they are represented in the original domain and the VILD domain ($I_{mn}^{\left(L\right)}\left[t\right],\psi_{mn}^{\left(L\right)}\left[t\right],\;t\;=\;30$). The compact VILD representation provides an excellent domain for feature extraction and recognition.

An interpretation of these results is given in Figure 12, where (first row) we show an original image $I_{mn}^{\left(L\right)}\left[t\right]$, its CHF output module $M_{mn}^{\left(L\right)}\left[t\right]$, and the thresholded phase $\psi_{mn}^{\left(L\right)}\left[t\right]$ (t=30), and then (second row) we highlight some details of the captured image as they appear in the original domain and the VILD domain ($I_{mn}^{\left(L\right)}\left[t\right],\psi_{mn}^{\left(L\right)}\left[t\right],\;t\;=\;30$). We recognize that the VILD domain highlights the contribution in structured areas only, implicitly performing a kind of background subtraction. Therefore, using the VILD-SLAM approach, variations occurring within non-structured areas are inherently rejected, making the approach noise resilient. This behavior is beneficial also in dynamic environments where a number of non-stationary features, e.g., illumination, change throughout the VSLAM.

A few remarks are in order. The VILD-SLAM is dynamically aware, in the sense that it represents the scene where noise is rejected; still, it does not explicitly account for moving object, and this is left for further study. To sum up, VILD-SLAM shows the potential to improve real-time state-of-the-art solutions. The ability to reject noise components suggests its integration within a deep learning-based system; this relevant point is out of the scope of the paper and it is left for further studies.

## 7. Conclusions

In conclusion, this work introduced VILD-SLAM, a novel approach for Visual Simultaneous Localization and Mapping that leverages Circular Harmonic Filters (CHFs) to improve trajectory estimation from stereo camera images. By transforming images into the Visual Localization Domain (VILD), the method enhances visually significant features such as edges and orientations while reducing the impact of noise and uniform regions. This transformation improves feature matching and tracking, addressing key challenges in localization and mapping tasks. The CHF filtering process provides both magnitude and phase information, enabling the extraction of critical features and refinement of orientation estimations. This approach reduces mean squared error (MSE) and mitigates scale drift (SD), leading to trajectory estimations closely aligned with ground-truth GPS data. Experimental evaluations on stereo datasets demonstrated that VILD-SLAM outperforms traditional spatial-domain methods, particularly in complex visual environments. While CHF filtering introduces some computational overhead, the significant improvements in accuracy validate the trade-off. Future work will explore integrating VILD as an input domain for deep neural networks to enhance learning-based SLAM systems. Additional efforts will focus on optimizing efficiency and incorporating multi-modal sensor data for broader applicability in diverse scenarios.

## Figures and Tables

**Table 2:** The table reports the mean square error (MSE [m^2^]), root mean square error (RMSE [m]), and scale drift (SD[]) of the VILD-SLAM based on the maps $\psi_{mn}^{\left(L\right)}\left[t\right],\psi_{mn}^{\left(R\right)}\left[t\right]$, for two parameter settings ($\theta \;=\;0.1,N_{M}\;=\;20$ and $\theta \;=\;0.08,N_{M}\;=\;5$). The metrics are provided for the complete path, as well as separately for the first and the second segment.

$\psi_{\mathrm{mn}}^{\left(L\right)}\left[t\right],\psi_{\mathrm{mn}}^{\left(R\right)}\left[t\right]$ Performances	$\theta \;=\;0.1,N_{M}\;=\;20$			$\theta \;=\;0.08,N_{M}\;=\;5$		
	Full Path	1° Segment	2° Segment	Full Path	1° Segment	2° Segment
MSE [m^2^]	23.1862	32.0529	7.7102	35.0658	44.6947	15.9963
RMSE [m]	4.8152	5.6615	2.7767	5.9216	6.6854	3.9995
SD	−0.0482	−0.0374	−0.0310	−0.0707	−0.0658	−0.0387

**Table 3:** Performance metrics (MSE [m^2^], RMSE [m]), and SD[]) achieved using the algorithm in [14] for the complete path and separately for the first and the second segment.

State-of-the-Art Performances [14]	Full Path	1° Segment	2° Segment
MSE [m^2^]	68.7747	82.7192	44.0547
RMSE [m]	8.2930	9.0950	6.6374
SD	0.8995	−0.1142	−0.0634

## Data Availability

The data used in this study are available in the public domain UTIAS Long-Term Localization and Mapping Dataset at http://asrl.utias.utoronto.ca/datasets/2020-vtr-dataset/.
